# Evaluating toxicity of Varroa mite (*Varroa destructor*)-active dsRNA to monarch butterfly (*Danaus plexippus*) larvae

**DOI:** 10.1371/journal.pone.0251884

**Published:** 2021-06-02

**Authors:** Niranjana Krishnan, Maura J. Hall, Richard L. Hellmich, Joel R. Coats, Steven P. Bradbury

**Affiliations:** 1 Toxicology Program and Department of Entomology, Iowa State University, Ames, Iowa, United States of America; 2 Corn Insects and Crop Genetics Research Unit, United States Department of Agriculture, Ames, Iowa, United States of America; 3 Department of Natural Resource Ecology and Management, Iowa State University, Ames, Iowa, United States of America; Ghent University, BELGIUM

## Abstract

Varroa mites (*Varroa destructor*) are parasitic mites that, combined with other factors, are contributing to high levels of honey bee (*Apis mellifera*) colony losses. A Varroa-active dsRNA was recently developed to control Varroa mites within honey bee brood cells. This dsRNA has 372 base pairs that are homologous to a sequence region within the Varroa mite calmodulin gene (*cam*). The Varroa-active dsRNA also shares a 21-base pair match with monarch butterfly (*Danaus plexippus*) calmodulin mRNA, raising the possibility of non-target effects if there is environmental exposure. We chronically exposed the entire monarch larval stage to common (*Asclepias syriaca*) and tropical (*Asclepias curassavica*) milkweed leaves treated with concentrations of Varroa-active dsRNA that are one- and ten-fold higher than those used to treat honey bee hives. This corresponded to concentrations of 0.025–0.041 and 0.211–0.282 mg/g leaf, respectively. Potassium arsenate and a previously designed monarch-active dsRNA with a 100% base pair match to the monarch *v-ATPase A* mRNA (leaf concentration was 0.020–0.034 mg/g) were used as positive controls. The Varroa mite and monarch-active dsRNA’s did not cause significant differences in larval mortality, larval or pupal development, pupal weights, or adult eclosion rates when compared to negative controls. Irrespective of control or dsRNA treatment, larvae that consumed approximately 7500 to 10,500-mg milkweed leaf within 10 to 12 days had the highest pupal weights. The lack of mortality and sublethal effects following dietary exposure to dsRNA with 21-base pair and 100% base pair match to mRNAs that correspond to regulatory genes suggest monarch mRNA may be refractory to silencing by dsRNA or monarch dsRNase may degrade dsRNA to a concentration that is insufficient to silence mRNA signaling.

## Introduction

Varroa mites are thought to be a significant stressor causing honey bee decline [1]. The mites attach to bees, transmit viruses, and consume the honey bees’ fat bodies and, to a lesser extent, hemolymph [2]. The fat body is integral for immune function, pesticide detoxification, hormone regulation, and enhanced overwintering survival [3]. Impairment of fat body function in a sufficient percentage of honey bees can contribute to colony declines [2]. Several control methods are used to reduce Varroa mite populations. Currently, the most effective and economical method is to employ chemical miticides [4]. In the U.S., there are currently 15 miticides approved for controlling Varroa mites in beehives [5]. Due to the heavy reliance on these products, Varroa mites have developed resistance to several compounds [6–9], primarily due to enhanced metabolism and/or target site insensitivity [10]. Three of the miticides for which there are no reported Varroa mite resistance—formic acid, oxalic acid, and thymol—could harm bees by inducing toxicity [11–14], causing stress [15], and affecting brood development [16–18]. Hence, there is a need to develop new miticides that specifically target Varroa mites without negatively affecting honey bees.

The development of double-stranded (dsRNA) insecticides creates the means to selectively target insect pest species. DsRNA insecticides employ RNA interference (RNAi) technology. RNAi is a mechanism whereby specific messenger RNA (mRNA) transcripts are targeted by small interfering RNAs (siRNAs) and silenced via nuclease activity or translational repression [19, 20]. RNAi can be used to design insecticides that specifically target pest species by identifying regions on the pest mRNA that have little or no overlap with mRNA of non-target species [21]. For example, silencing critical genes in several pest insect species did not results in adverse effects across a range of taxonomically dissimilar non-target species [22, 23]. It has been hypothesized that a dsRNA could be efficacious only if it shares a minimum sequence of 19–21 nucleotides with the target insect mRNA [22–24].

Bayer Crop Science has developed a dsRNA to control Varroa mites (*Varroa destructor*) within honey bee (*Aphis mellifera*) brood cells [25] (see S1 Fig). This dsRNA has 372 base pairs that are homologous to a sequence region within the Varroa mite calmodulin gene (*cam*) [S2 Fig]. This gene encodes calmodulin (CaM), which is an essential calcium-binding protein that regulates multiple protein targets. The prototype product is formulated as an 80% sucrose solution that is placed in the hive. Nurse bees consume the dsRNA sucrose solution and deliver it to the brood cells. The mites could be exposed to the dsRNA through contact with the sucrose solution deposited by adult honey bees, brood food made with the 80% sucrose solution, and/or through consumption of larval or adult hemolymph [26].

The Varroa dsRNA has a 99% nucleotide match to the Varroa mite calmodulin mRNA (S2 Fig) and a 74% nucleotide match, which includes a contiguous sequence of 14 nucleotides, to the honey bee calmodulin mRNA. There are no contiguous 21-nucleotide overlaps between the Varroa-active dsRNA and the honey bee genome (S3 Fig). Previous studies have shown that honey bees are mostly insensitive to orally delivered dsRNA [27], including dsRNA molecules that have a 100% sequence match to their mRNA [28].

Exposure of the Varroa-active dsRNA product to non-target insects outside the hive, including monarch butterfly (*Danaus plexippus*) larvae, is highly unlikely and supports a low environmental risk determination. However, the Varroa-active dsRNA has a 21-base pair match to monarch calmodulin mRNA (S4 Fig). Since dsRNA orthologs could be efficacious against insect mRNA if they share a sequence length of at least 19 to 21 nucleotides [22–24], the potential hazard to monarch larvae, if they are exposed to the Varroa-active dsRNA, cannot be precluded.

To assess risks of dsRNA insecticides to non-target arthropod species, the United States Environmental Protection Agency (USEPA) uses a four-tiered testing scheme based on the microbial pesticide data requirements published under 40 CFR 158.2150 and the associated OCSPP Harmonized Guidelines 885 and 850 series [29, 30]. Tier I studies are designed to estimate hazards to several non-target arthropod taxa under exposure concentrations several times higher than the highest concentrations (≥ 10X when possible) expected to occur under realistic field exposure scenarios. A lack of adverse responses under these exposure conditions, presumably, provide sufficient certainty that there would not be unreasonable effects to the environment if the product were registered, i.e., complex, higher Tier testing with realistic exposure levels is not required.

Previous research by Pan et al. [31] explored the extent to which neonate monarch larvae are sensitive to monarch and western corn rootworm specific dsRNAs that target the *v-ATPase A* mRNA following a two-day dietary exposure (5 mg/mL of respective dsRNAs solutions applied to 0.5 cm diameter honeyvine milkweed [*Cynanchum laeve*] leaf discs). V-ATPase A is a proton pump that maintains pH equilibrium at the cellular and organismal level and plays an important role in cellular function by interacting with a variety of proteins [32]. Given V-ATPase A’s essential physiological function, it was expected monarch *v-ATPase A* mRNA would be silenced by the monarch-active dsRNA, and potentially, the western corn rootworm dsRNA as it shares a high sequence similarity with the monarch mRNA. In turn, silencing monarch *v-ATPase A* mRNA should result in reduced growth leading to a high level of larval mortality [33]; however, Pan et al. [31] reported no adverse effects for either dsRNA. The lack of adverse effects to the rootworm- and monarch-active dsRNA could be due to a short dietary exposure period that may have resulted in an insufficient internal dose and/or a peak internal dose that did not overlap with key development events (i.e., larval molts, pupal formation, and/or adult eclosion).

In the present paper, we expand our understanding of non-target effects of dsRNA insecticides by undertaking chronic dietary studies with the Varroa calmodulin dsRNA, which has a 21-nucleotide overlap with the monarch calmodulin mRNA, and monarch v-ATPase A dsRNA, which is assumed to have a 100% nucleotide match with the monarch *v-ATPase* mRNA [31]. We assessed chronic toxicity of Varroa-active dsRNA to monarch larvae by exposing them for approximately two weeks to concentrations 10-fold greater than would be expected if the formulated product were inadvertently applied to milkweed. Given the shared nucleotide sequence, we hypothesized that continuous dietary exposure of the Varroa and monarch-active dsRNA through the entire larval stage could adversely affect larval survival and growth; instar and pupal development; and/or eclosion of adult monarch butterflies.

## Materials and methods

### Rearing monarch butterflies and milkweed

Monarch butterfly eggs for four of the six bioassay runs were obtained from the 2016 colony maintained by the U.S. Department of Agriculture (USDA), Corn Insects and Crop Genetics Research Unit in Ames, Iowa (see [34]). The fifth and sixth bioassay runs were conducted using eggs obtained from a colony maintained by the University of Kansas (Dr Orley Taylor, Director of Monarch Watch). The first three bioassays were undertaken on common milkweed (*Asclepias syriaca*), a native species found in U.S. Midwestern states, using the Iowa monarchs. To see if a different milkweed species and/or a source of monarchs influenced sensitivity to dsRNA, the last three bioassays (one with Iowa monarchs and two with Kansas monarchs) were conducted on tropical milkweed (*Asclepias curassavica*).

Young, non-senescent common milkweed leaves were collected from a restored prairie in Ames, Iowa, in September and October of 2018. Tropical milkweed leaves were reared in Iowa State University greenhouses as previously reported [34]. All milkweed leaves were washed with 10% bleach solution and rinsed three times with water before use. Leaves were dried using a salad colander and WypAll wiper tissues (Kimberly-Clark Professional) prior to use in the bioassays.

### Chemicals employed and preparation of treatment solutions

A 64 mg/mL aqueous solution of Varroa-active dsRNA (lot number: STG4-0038) was provided by Bayer Crop Science. The prototype dsRNA formulation contains 2.1 mg/mL Varroa-active dsRNA in an 80% sucrose solution (J. Fischer, personal communication). In a preliminary assay, we provided fifth-instar monarchs common milkweed leaves coated with an 80% sucrose aqueous solution (a formulation blank). The larvae did not consume the treated leaves. Consequently, we prepared 2.1 mg/mL (1X environmental concentration) and 21 mg/mL (10X concentration) Varroa-active dsRNA solutions for bioassays by diluting the 64 mg/mL stock solution in deionized water, rather than a sucrose solution.

Bayer also synthesized and provided a 25.4-mg/mL aqueous solution of monarch butterfly dsRNA (batch number: M1166) with a 100% base pair match to the monarch *v-ATPase A* mRNA. This monarch-active dsRNA was synthesized from previously designed forward and reverse primers [31]. The monarch V-ATPase A dsRNA was selected as a putative positive dsRNA control. We prepared a 5-mg/mL monarch-active dsRNA solution in deionized water, which is the same concentration used by Pan et al. [31] in their monarch bioassays with neonates.

Potassium arsenate (CAS number: 7784-41-0; Lot number: SLBN3865V), purchased from Sigma Aldrich, also was used as a positive control. We used an aqueous concentration of 1 mg/mL in the bioassays, which corresponded to the LC_100_ based on a preliminary assay in which larvae were fed treated tropical milkweed leaves.

### Toxicity bioassays

Toxicity bioassay studies were conducted at 24 to 27°C and 45 to 65% relative humidity, with a 16:8 light: dark cycle. Both common and tropical milkweed bioassays employed six treatments: untreated leaves, deionized water-treated leaves, potassium arsenate-treated leaves, monarch-active dsRNA-treated leaves, and Varroa-active dsRNA-treated leaves at two nominal concentrations of 2.1 and 21 mg/mL. Fifteen and 10 larvae were used per treatment group in the common and tropical milkweed bioassays, respectively. Both milkweed bioassays were conducted three times, with each run employing a different larval generation. Thus, 45 and 30 larvae were employed per treatment group (n = 6) in the common and tropical milkweed bioassays, respectively. Water, monarch-active dsRNA, and the Varroa-active dsRNA solutions were applied using a 59-mL fingertip sprayer bottle (Equate brand). Both sides of the leaves were sprayed with the solutions (multiple sprays were carried out for bigger leaves) and manually spread across the leaf surface using clean nitrile gloves (VWR International), as needed, to ensure complete coating. The leaves were then hung on a wire and clamped with paper clips until dry (10 to 20 minutes). The potassium arsenate solution was applied on one side of the leaf using a micropipette (20 to 30 μL was spread over a 250 mg leaf). These leaves were placed on a tray with absorbent bench paper and allowed to dry.

Monarch larvae were reared according to methods described Krishnan et al. [34]. Neonates were plated on a treated or untreated leaf (220 to 280 mg) in individual petri plates (60 mm x 15 mm containing a thin layer of 2% agar: water) using a paintbrush. Freshly treated (1 or 10X Varroa-active dsRNA, monarch-active dsRNA, or deionized water) or untreated leaves were provided every two days for the first six to eight days of a bioassay, and daily thereafter. Increasing leaf mass (up to 2700 to 3300 mg per day) was provided as the larvae developed. Every 24 hours, larval mortality, abnormal behavior, and leaf consumption (i.e., minimal consumption vs. consumption of most or entire leaf mass provided) were recorded. Instar was recorded every 96 hours. Days to pupation, pupal weights, and adult eclosion (i.e., adult emergence) were recorded for the surviving larvae. Results were analyzed from individual bioassays where both the negative controls (larvae fed untreated and water-treated leaves) produced less than 35% mortality from neonate to pupation. This upper bound control mortality was based on a maximum control mortality of 30% in 96-hour monarch larval dietary bioassays (see [34]).

Three times during each bioassay, three additional leaf samples (mass range: 221 to 2192 mg) were randomly treated with water or one of the three dsRNA solutions. These leaves were allowed to dry, then were wrapped in aluminum foil and stored in Ziploc^®^ bags at -20°C for QuantiGene analysis.

### Sample extraction and processing

Prior to RNA extraction from treated leaves, the laboratory bench was wiped with RnaseZap to ensure an RNAase-free environment. Each frozen leaf sample was weighed and placed in a mortar with a small amount of liquid nitrogen. Each sample was ground, and the resultant powder was transferred to a pre-chilled phase lock gel tube (Qiagen, Catalog# 129065 & 129073). One mL of TRIzol (Ambion Life Technologies) was added per 0.1 g of leaf tissue. Samples were vortexed for three minutes and then incubated at room temperature (RT) for one hour. Chloroform (Fisher Scientific) was then added to the samples (0.3 mL for every mL of TRIzol). Samples were vortexed again for one minute and incubated at RT for 10 minutes. Samples were then centrifuged at 9000 Relative Centrifugal Force (RCF) at 2 to 6°C. The upper aqueous phase was transferred to a 15-mL falcon tube. The RNA was precipitated by adding 0.5 mL of isopropyl alcohol (Fisher Scientific) per ml of supernatant. The solutions were then mixed by inverting the tubes multiple times. Samples were stored in either a -20°C or -80°C freezer for 0.5 to 24 hours, and then centrifuged at 9000 RCF for 15 to 20 minutes at 2 to 6°C. The supernatant was discarded, and the RNA pellet was washed with ~5 ml of 70% ethanol prepared in nuclease-free Ultrapure Distilled Water (Invitrogen Lot#2063810). The pellets were then centrifuged at ~9000 RCF for 10 minutes at 2 to 6°C, and the supernatant was discarded. Another centrifugation at ~9000 RCF for one minute at 2 to 6°C was conducted, and the residual liquid was removed with a pipette. The RNA pellets were briefly air dried (≤10 minutes) and dissolved in an appropriate volume of nuclease-free Ultrapure Distilled Water (100 to 250 μL per gram of starting tissue). The RNA was stored in a –20°C or –80°C freezer until quantification. Prior to QuantiGene analysis, each milkweed leaf extract was normalized with sample diluent to fall within the standard curve.

### QuantiGene analysis

Total extracted RNA was quantified using a QuantiGene^®^ (QG) 2.0 Singleplex assay kit (Invitrogen Ref#13216). To begin, 1.2 mL of a custom QuantiGene probe set was combined with 90 μL of the appropriate sample (water background control, reference standards, or the test samples) in a disposable PCR plate. The custom probes were designed by the manufacturer to hybridize to the specific dsRNA sequences used in this study. Separate probes were used for Varroa-active dsRNA and monarch butterfly dsRNA samples. After the addition of all standards and samples, the denaturing plate was sealed with plate foil (ThermoFisher Ref#AB0626) and heated at 98°C (±5°C) for 5 minutes and subsequently held at 55°C (±5°C) for 30 minutes.

A premixed QG 2.0 working solution was prepared by adding nuclease-free water, lysis mixture, and blocking reagent. Eighty μL of QG 2.0 working solution was added to each well of the assay plate. For each well containing 80 μL of denatured standard/sample in the denaturing plate, 20 μL was plated into the wells of the assay plate in triplicate. This resulted in 80 μL QG 2.0 working solution and 20 μL denatured standard/sample per assay plate well. The plate was sealed with foil and incubated at 55°C (±5°C) for 16 to 24 hours.

After overnight hybridization, the wells of each plate were washed three times with 300 μL of QG 2.0 Wash Buffer. The plates were then inverted and tapped to dry. One hundred μL of preamplifier solution was added to each well; plates were then sealed with a plate foil and incubated at 55°C (±5°C) for 55 to 65 minutes. The previous step was repeated for the amplifier solution and the label probe solution. QuantiGene solutions were prepared following the manufacturer’s recommendations and are outlined in S1 Table. Following incubation with the label probe solution, the plates were washed three times with 300 μL/well of QG 2.0 Wash Buffer and allowed to dry for no more than five minutes.

After the last washes, 100 μL of QG 2.0 Substrate was added to each well and the plate was sealed with foil and incubated for 5 to 15 minutes at room temperature. The median luminescence of each well was captured by a Synergy-HTX Multi-mode Microplate Reader (BioTek). The concentrations of Varroa-active dsRNA and monarch-active dsRNA were calculated from a standard curve fit with a 4‐parameter logistic regression model (S5 Fig). Each sample was run in triplicate, and the mean concentrations were calculated.

### Statistical methods

**Fig 1:**
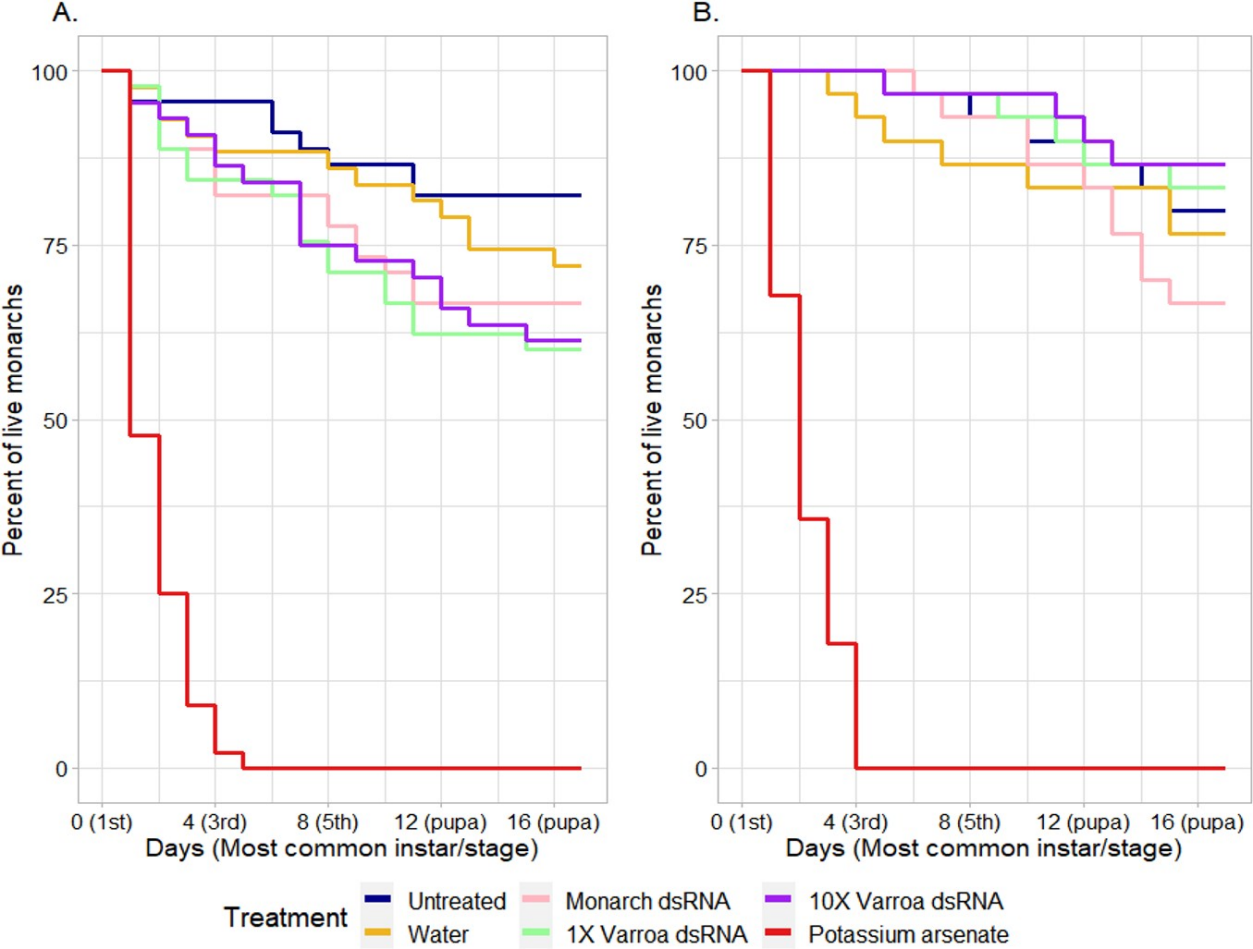
Monarch mean percent mortality over time, from neonate larvae to pupae, with data combined over all bioassay runs. Larvae were fed common (A) or tropical (B) milkweed leaves that were untreated (UN), treated with deionized water (WT), 5 mg/mL monarch-active dsRNA solution (MB), 2.1 (VL) and 21 (VH) mg/mL Varroa-active dsRNA solutions, or 1 mg/mL potassium arsenate solution (KA). Missing larvae (including 1 larva that was accidentally killed and five that went missing) were excluded from analysis.

All statistical analyses were done in RStudio 1.1.383 (R version 3.5.2). Common and tropical milkweed bioassay results were analyzed independently. In both milkweed species, potassium arsenate treatments (positive control) caused 100% larval mortality within five days (Fig 1) and were excluded from analyses. Generalized linear models (glm) accounted for both run (three bioassay runs each for common and tropical milkweed) and treatment effects. There was no run-by-treatment interaction (p > 0.05); consequently, the following equation was used: response ~ run + treatment.

To analyze larval mortality (larvae alive/larvae dead) and adult eclosion (adults emerged/adults not emerged), we fit a binomial or a quasibinomial (to account for overdispersion) glm model and used type 3 ANOVA (obtained from the “car” package) to look for differences between treatments. A quasipoisson (to account for underdispersion) glm model and type 3 ANOVA were used to evaluate days from neonate to pupation. Following the removal of a single outlier in the common milkweed water treatment (this pupa’s weight was one-third the weight of an average pupa in the same treatment group), the residual plots for the pupal weights showed the data were normally distributed and had homogenous variances. Consequently, a gaussian glm model and type 3 ANOVA were used to evaluate differences in pupal weights between treatments. If significant treatment or run effects were identified (p < 0.05), Dunnett’s test for multiple comparisons (emmeans package) was used to compare the control response to the insecticide treatment responses.

## Results

### Sample extraction and QuantiGene analysis

In the common milkweed bioassays, a subset of two leaves from each treatment group (5 mg/mL monarch-active dsRNA and 2.1 and 21 mg/mL Varroa-active dsRNA) and bioassay run were analyzed. Measured concentrations for 2.1 (1X) and 21 (10X) mg/mL Varroa-active dsRNA ranged from 0.013 to 0.032 and 0.144 to 0.389 mg/g, respectively. The measured concentration of monarch-active dsRNA ranged from 0.020 to 0.021 mg/g (Table 1).

**Table 1:** The mean concentration measured for each treatment group and the overall mean.

Milkweed species	Treatment	Concentration dsRNA (mg/g)			
		Run 1^a^	Run 2^a^	Run 3^a^	Overall^b^
**Common milkweed**	Monarch	0.020 (± 0.005)	0.020 (± 0.015)	0.021 (± 0.015)	0.020 (± 0.0004)
	1X Varroa	0.013 (± 0.003)	0.030 (± 0.018)	0.032 (± 0.014)	0.025 (± 0.009)
	10X Varroa	0.389 (± 0.32)	0.144 (± 0.138)	0.312 (± 0.274)	0.282 (± 0.102)
**Tropical milkweed**	Monarch	0.036 (± 0.005)	0.037 (± 0.014)	0.030 (± 0.016)	0.034 (± 0.003)
	1X Varroa	0.020 (± 0.013)	0.065 (± 0.049)	0.036 (± 0.021)	0.041 (± 0.019)
	10X Varroa	0.316 (± 0.062)	0.143 (± 0.036)	0.173 (± 0.091)	0.211 (± 0.075)

^a^ The mean dsRNA concentration and standard deviation (SD) per designated bioassay run.

^b^ The mean dsRNA concentration and standard deviation (SD) over all bioassay runs.

Monarch-active dsRNA = 5 mg/mL monarch-active dsRNA solution concentration; 1X and 10X Varroa-active dsRNA = 2.1 and 21 mg/mL Varroa-active dsRNA solution concentrations, respectively.

In the tropical milkweed bioassays, a subset of two to three leaves for each treatment group and run were analyzed. Measured concentrations for 2.1 and 21 mg/mL Varroa-active dsRNA ranged from 0.020 to 0.065 and 0.143 and 0.316 mg/g, respectively. The measured concentration of monarch-active dsRNA ranged from 0.030 to 0.037 mg/g (Table 1). The 21 mg/mL treatment was 2- to 16-fold higher and 5- to 30-fold higher than the 2.1 mg/mL treatment in the tropical and common milkweed bioassays, respectively.

### Toxicity bioassays

In the tropical milkweed bioassays, larvae provided untreated, water-treated, 5 mg/mL monarch-active dsRNA-treated, and 2.1 and 21 mg/mL Varroa-active dsRNA-treated tropical milkweed leaves had 20 (± 10), 23 (± 6), 33 (± 21), 17 (± 21), and 13 (± 6) mean (± SD) percent mortality, respectively; no noticeable difference in toxicity was seen between Iowa and Kansas colony larvae. In the common milkweed bioassays, the same treatments caused 18 (± 10), 27 (± 10), 33 (± 7), 40 (± 20), and 39 (± 12) mean percent mortality, respectively from neonate to pupation (Table 2). When Abbott’s formula was used to account for mortality in the untreated control group, the average larval percent mortality rates in the water, monarch-active, 1X Varroa-active, and 10X Varroa-active treatment groups ranged from 4–11%, 16–18%, 0–27%, and 0–26%, respectively, when considering both common and tropical milkweed bioassays. While mortality occurred over multiple days for all treatments (excluding potassium arsenate, which killed all treated larvae within five days), there were some temporal trends in mortality. In the common milkweed bioassays, a greater proportion of larval mortality in the negative controls and dsRNA groups occurred in the first eight days; the opposite was true in the tropical milkweed bioassays (Fig 1).

**Table 2:** Monarch larval percent mortality following treatment with Varroa-active dsRNA and two positive and two negative controls^a^.

Milkweed species (# of larvae treated)	Treatment	Larval percent mortality^b^				
		Run 1	Run 2	Run 3	Mean (± SD)^c^	Mean corrected mortality^d^
**Common milkweed**	Untreated	20	7	27	18 (± 10)	0
	Water	33	15	33	27 (± 10)	11
	Monarch-active dsRNA	27	40	33	33 (± 7)	18
	1X Varroa-active dsRNA	40	60	20	40 (± 20)	27
	10X Varroa-active dsRNA	27	50	40	39 (± 12)	26
	Potassium arsenate	100	100	100	100 (± 0)	100
**Tropical milkweed**	Untreated	10	20	30	20 (± 10)	0
	Water	30	20	20	23 (± 6)	4
	Monarch-active dsRNA	10	40	50	33 (± 21)	16
	1X Varroa-active dsRNA	10	40	0	17 (± 21)	0
	10X Varroa-active dsRNA	10	20	10	13 (± 6)	0
	Potassium arsenate	100	100	100	100 (± 0)	100

^a^ Monarch larvae were fed untreated leaves and leaves treated with deionized water, 5 mg/mL monarch-active dsRNA solution, 2.1 (1X) and 21 (10X) mg/mL Varroa-active dsRNA solutions, and 1 mg/mL potassium arsenate solution. All solutions were made in deionized water.

^b^ The percentage of larvae that died from neonate to pupation in each bioassay run. Six missing larvae (including one accidental death) over all treatments were excluded from analyses.

^c^ The mean larval percent mortality and standard deviation (SD) over all bioassay runs.

^d^ Abbott’s formula was employed to correct for untreated control mortality. Corrected percent mortality = [1- (number of larvae surviving in treatment group ÷ number of larvae surviving in untreated control group)] x 100.

In general, across all assays, the rates of mortality in dsRNA groups were similar to those observed in the two negative control groups. In both the tropical and common milkweed bioassays, there were no significant differences in larval mortality between treatment groups (χ^2^ = 4.18; df = 4; p = 0.382 and χ^2^ = 6.89; df = 4; p = 0.142, respectively). Combined mortality data from both milkweed species also was not different (χ^2^ = 4.97; df = 4; p = 0.290).

**Fig 2:**
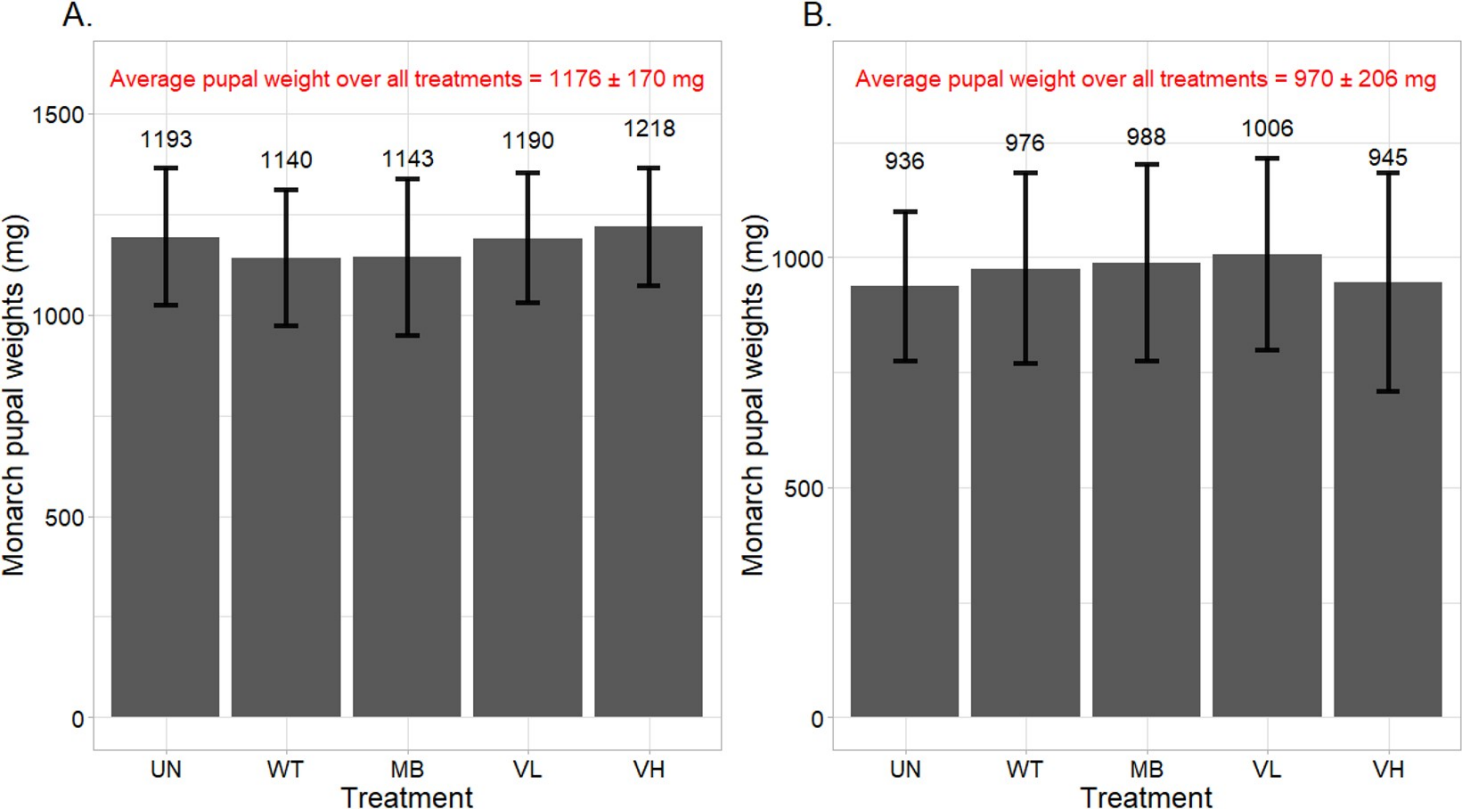
Average monarch pupal weight (in mg) in each treatment (data combined over all bioassay runs). Larvae were fed common (A) or tropical (B) milkweed leaves that were untreated (UN), treated with deionized water (WT), 5 mg/mL monarch-active dsRNA solution (MB), or 2.1 (VL) and 21 (VH) mg/mL Varroa-active dsRNA solutions. Bars represent the mean ± one standard deviation. A single pupa in the common milkweed water treatment was excluded from analyses.

With both milkweed species, the monarch and Varroa-active dsRNA treatments did not delay larval development from first through fifth instar and fifth instar to pupae (Table 3). The mean (± SD) developmental time from neonate to pupae ranged from 11.2 (± 0.95) to 11.6 (± 1.1) days with common milkweed, with no differences between treatment groups (χ^2^ = 1.44; df = 4; p = 0.838). For tropical milkweed, developmental times ranged from 11.2 (± 0.67) to 11.5 (± 1.2) days (χ^2^ = 4.96; df = 4; p = 0.292). Larvae took 10 to 15 days to pupate, with a median of 11 days in all instances. Mean (± SD) monarch pupal weights between treatments in the common and tropical milkweed bioassays ranged from 1140 (± 168) to 1218 (± 145) mg and 936 (± 162) to 1006 (± 208) mg, respectively (Fig 2). There were no differences in pupal weights between groups for both milkweed species (F = 1.36; df = 4; p = 0.250 and F = 0.521; df = 4; p = 0.721 for common and tropical milkweed, respectively). The inclusion of a single outlier in the common milkweed water treatment did not change the results (F = 1.75; df = 4; p = 0.142).

**Table 3:** Monarch larval development following treatment with Varroa-active dsRNA and one positive and two negative controls^a^.

Milkweed species (# of larvae treated)	Treatment	% of monarch instar/stage observed over all bioassay runs^b^			Mean (± SD) days to pupae^c^
		Day 4: Third instar	Day 8: Fifth instar	Day 12: Pupae	
**Common milkweed**	UN	57	86	86	11.2 (± 1.0)
	WT	68	68	87	11.6 (± 1.1)
	MB	57	87	93	11.2 (± 0.95)
	VL	70	67	93	11.3 (± 0.88)
	VH	63	78	93	11.3 (± 0.96)
**Tropical milkweed**	UN	63	92	92	11.5 (± 0.88)
	WT	70	87	83	11.5 (± 1.2)
	MB	55	90	100	11.2 (± 0.67)
	VL	64	84	84	11.4 (± 1.1)
	VH	65	92	85	11.4 (± 1.0)

^a^ Monarch larvae were fed untreated leaves (UN) and leaves treated with deionized water (WT), 5 mg/mL monarch-active dsRNA solution (MB), and 2.1 (VL) and 21 (VH) mg/mL Varroa-active dsRNA solutions. All solutions were made in deionized water. Only data from larvae that successfully pupated were analyzed. Data were combined over all bioassay runs.

^b^ The percentage of surviving monarchs in a treatment that belonged to the third instar (Day 4), fifth instar (Day 8) and pupa (Day 12). Larvae that were molting to a new instar were considered to have molted on the same day.

^c^ The mean [and corresponding standard deviation (SD)] number of days it took surviving larvae in each treatment to form pupae. Larvae that were in “J” form were considered to have pupated on the same day.

**Fig 3:**
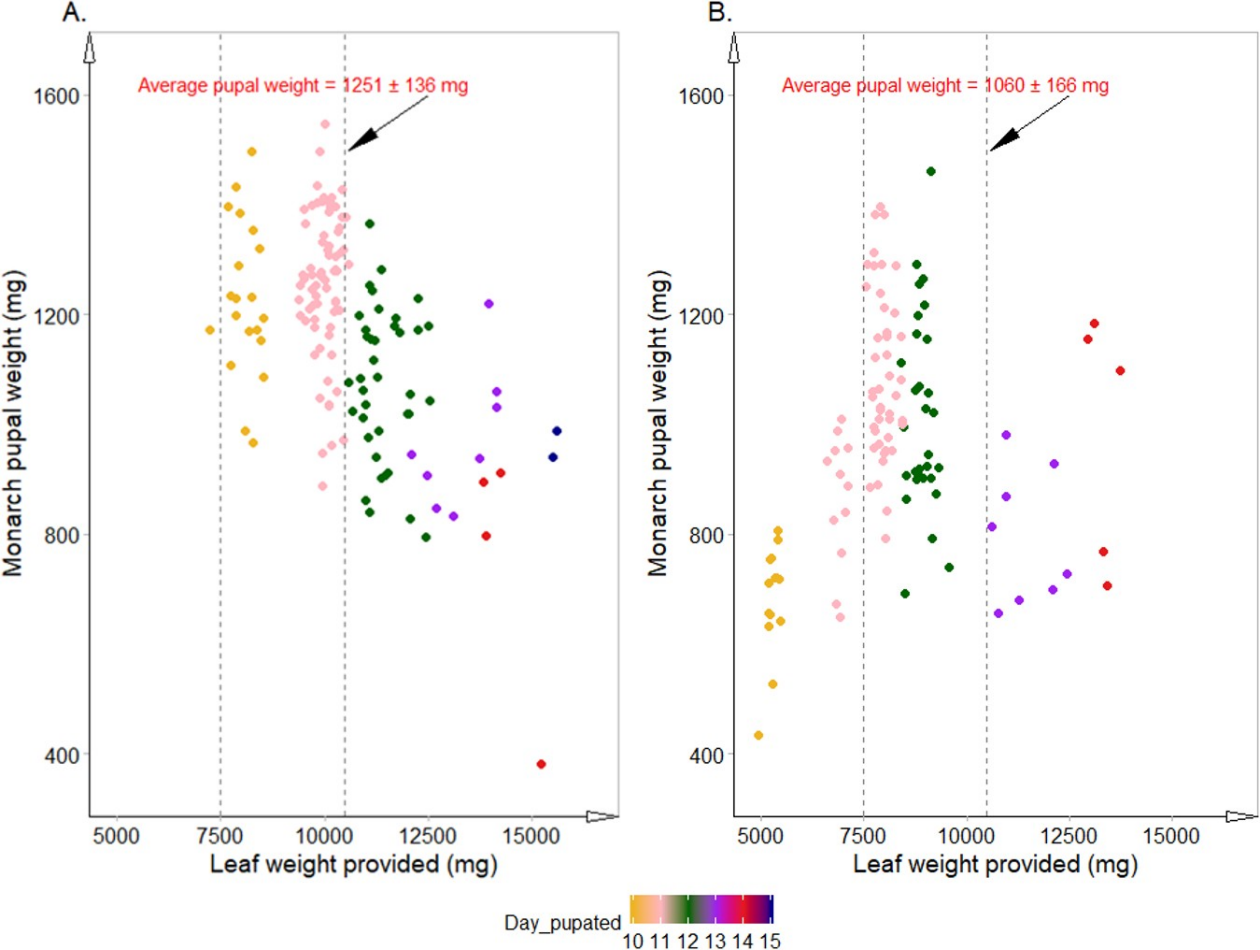
Individual monarch pupal weights (mg) plotted against individual weights (mg) of common (A) and tropical (B) milkweed leaf provided to each larva. Data were combined over all treatments and bioassay runs. The different colored dots represent the range of days it took the monarchs to pupate (see legend). The vertical dotted lines bound monarch pupae that were provided 7500 and 10,500 mg of milkweed leaf. The average weights of these pupae are provided.

Larvae that pupated within 10–11 days in the common milkweed bioassays and within 11–12 days in the tropical milkweed bioassays generally consumed between 7500 to 10,500 mg fresh leaves after reaching the third instar. These larvae generally had higher pupal weights (Fig 3). In one of the tropical milkweed bioassays, fewer than 7 g of milkweed leaf tissue were provided to larvae that had pupated on the tenth day—these pupae were smaller (Fig 3B). Larvae that did not pupate within 12 and 13 days in the common and tropical milkweed bioassays, respectively, did not consume most of the provided leaves. Thus, even though these larvae were provided a greater mass of leaves (freshly treated leaves were provided daily starting on or about Day 9), their pupal weights were often similar or lower than the pupal weights of larvae that pupated earlier.

There was, however, a significant difference in pupal development time and pupal weights between bioassay runs (p = 5.4 x 10^−10^ and 1.3 x 10^−3^, respectively, for common milkweed and p = 7.2 x 10^−4^ and 6.3 x 10^−4^, respectively, for tropical milkweed). In the common milkweed bioassays, the third bioassay run differed from the first two. The milkweed leaves in the third run had started to senesce, and the larvae took longer to feed on the poorer quality leaves and pupate (12.2 days vs. 11.3 days for each of the first two runs). The quality of the leaves also could have resulted in the significantly lower pupal weights (1111 mg vs. 1215 and 1213 mg in the first two runs), even though individual larvae in each run were provided a minimum of 7500 mg of leaf and the average leaf mass provided across runs was similar (range was 10,100 to 11,000 mg). In the tropical milkweed bioassays, individual larvae in the first run were provided fewer leaves on average (~7000 mg milkweed vs. ~9000 mg milkweed in the other two runs). The lack of sufficient leaf mass might have triggered pupation at a slightly earlier time (average was 11 days vs. 11.8 and 11.5 days for the last two runs) and also resulted in lower average pupal weights (897 mg vs. 942 and 1068 mg in the second and third bioassay run, respectively). Though larvae in the second and third bioassay runs were provided similar leaf mass, pupae from the second run were also significantly smaller (p = 0.015). These analyses show that, under the environmental conditions tested, monarch larvae need at least 7500 mg of fresh milkweed leaf in the first 10–11 days to reach a healthy pupal weight.

**Fig 4:**
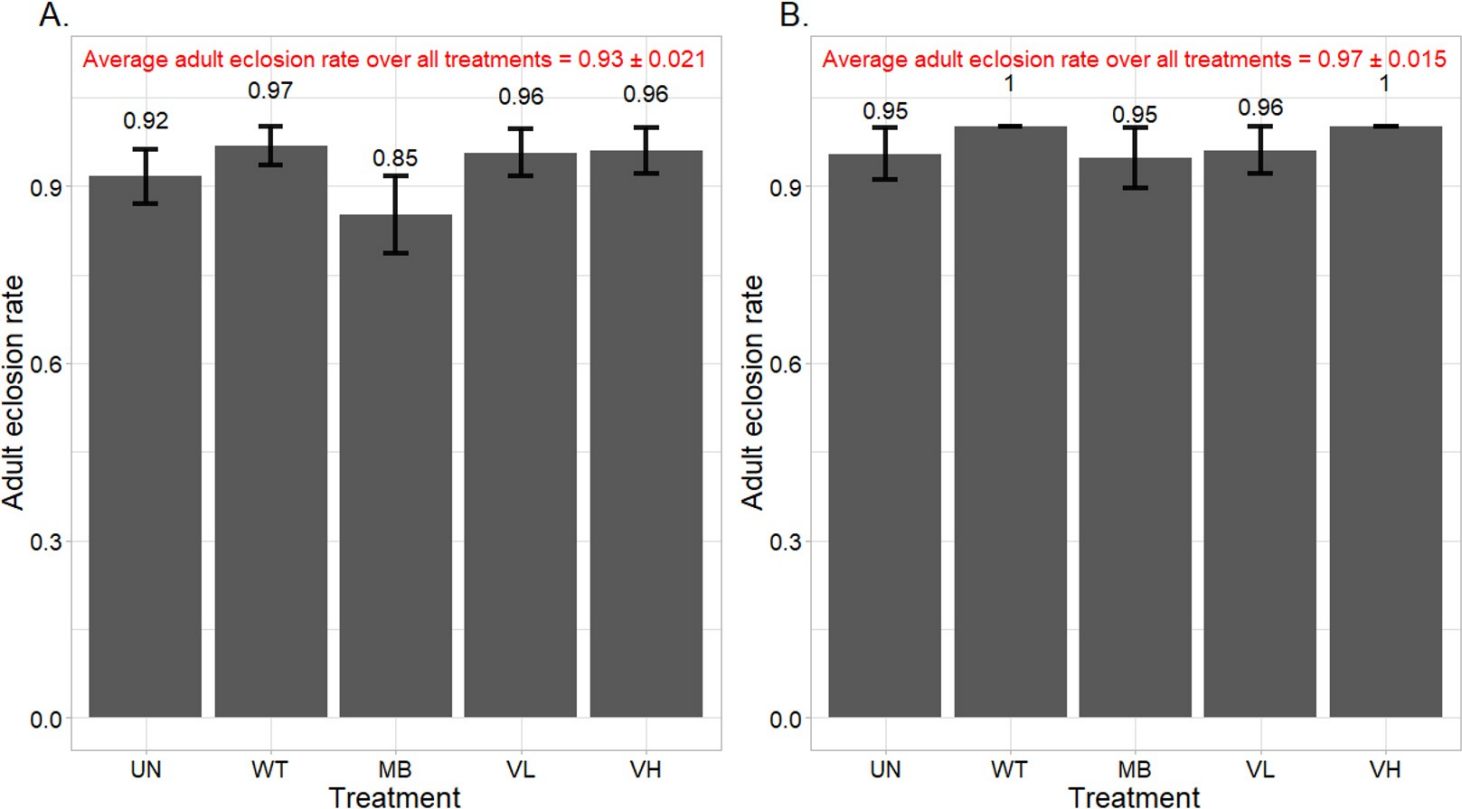
Average monarch adult eclosion rates of uninfected pupae in each treatment (data combined over all bioassay runs). Larvae were fed common (A) or tropical (B) milkweed leaves that were untreated (UN), treated with deionized water (WT), 5 mg/mL monarch-active dsRNA solution (MB), or 2.1 (VL) and 21 (VH) mg/mL Varroa-active dsRNA solutions. Bars represent the mean ± standard error.

In the first two common milkweed bioassays and the first tropical milkweed bioassay, there were low levels of bacterial infection in the pupae that suppressed adult eclosion rates (the overall infection rate in any of the treatment groups did not exceed 15%). These pupae were excluded from eclosion analyses but were included in the other analyses as the infection had no effect on the other measured endpoints. The mean (± SE) eclosion rate of uninfected pupae ranged from 0.85 (± 0.07) to 0.97 (± 0.03) and 0.95 (± 0.05) to 1.0 (± 0.0) in common and tropical milkweed bioassays, respectively (Fig 4). Again, there were no treatment differences in either milkweed species (χ^2^ = 7.07; df = 4; p = 0.132 and χ^2^ = 3.57; df = 4; p = 0.467 for common and tropical milkweed, respectively).

## Discussion

Some studies have hypothesized that a dsRNA that shares a minimum sequence of 19–21 nucleotides with an insect mRNA could cause mortality or adverse sublethal effects [22–24]. Hence, we expected chronic larval exposure to Varroa-active dsRNA and monarch-active dsRNA would cause high rates of mortality and sublethal effects; however, we observed no significant adverse effects. These results suggest bioinformatic analyses (e.g., 21 base pair matches) alone cannot predict potential dsRNA sensitivity to target species (and insensitivity to non-target species). Other factors including refractory genes, presence of high levels of dsRNase, and exposure to low environmental concentrations may prevent RNAi-mediated effects [23, 35, 36].

In the present study, monarch larvae were chronically exposed to nominal environmental concentrations of a Varroa-active dsRNA one to ten times greater than what would be applied in honey bee hives to control Varroa mites. Quantification of dsRNA concentrations on treated common and tropical milkweed leaves indicated mean leaf concentrations of 0.025 to 0.041 (1X treatment) and 0.211 to 0.282 mg/g leaf (10X treatment). In the common milkweed bioassays, overall larval mortality was higher in the Varroa-active dsRNA treatments (ca. 40%) compared to untreated (ca. 20%) and water-treated controls (ca. 30%), but the differences in toxicity were not statistically significant. The higher mortality in water and Varroa-active dsRNA treatments could have been caused by water retention in common milkweed. Common milkweed leaves are thick and even if their surfaces are air-dried following treatment, water within the leaves may not completely evaporate. Increased internal water content could reduce the nutritional value of the leaves and lead to slightly increased, but statistically insignificant, larval mortality. In the tropical milkweed bioassays, higher larval mortality was seen in the negative controls (ca. 20% for untreated and water-treated leaves) than in the 2.1 and 21 mg/mL Varroa-active dsRNA solutions (ca. 15%), however, this too was statistically insignificant. These mortality rates are also consistent with the historical control mortality rate of the Iowa State University monarch butterfly colony, which is 20 to 25% from neonate to pupa.

There were also no significant differences when mortality was averaged across both milkweed species. While monarch-active dsRNA-treated leaves had the highest combined mortality (33% vs 30% for Varroa treatments and 22% for control treatments), its effect on mortality was also not significant. The average larval mortality, when combined across milkweed species and control and dsRNA treatments was 27%. Given the historical morality rate and comparisons of mortality rates between control and dsRNA-treated leaves, the Varroa-active dsRNA at a dietary concentration 10X higher than would be expected in the environment is essentially non-toxic. Finally, larvae feeding on tropical and common milkweed had similar responses to dsRNA treatment, suggesting that different levels of cardenolides in common and tropical milkweed [37] seemingly do not alter the toxicity of dsRNA molecules through differential metabolic capability of the larvae.

Findings with Varroa-active dsRNA could indicate more than 21 base pair matches are required to elicit adverse effects. The monarch-active dsRNA, having a 100% match with monarch mRNA, was expected to serve as positive control; however, we observed only a marginal, non-significant, increase in mortality. To ascertain if individual cohorts of larvae were uniquely resistant to stomach poisons, we employed potassium arsenate as a positive control with each dsRNA bioassay. A 1 mg/mL solution consistently killed all larvae within 5 days.

There was no correlation between measured leaf concentration and average mortality rate for any of the treatments (p ≥ 0.19; S6 Fig). Across common and tropical milkweed bioassays, we observed up to a 3.3-fold difference in measured dsRNA concentrations for replicates across dsRNA treatments. Across both milkweed species, the average dsRNA leaf concentrations for the 5 mg/mL monarch-active dsRNA and the 2.1 and 21 mg/mL Varroa-active dsRNA treatments were 0.027, 0.033, and 0.246 mg/g leaf, respectively. Assuming a monarch larva consumed approximately 7500 mg of milkweed leaf tissue, we estimate internal doses of 0.20 mg of monarch-active dsRNA and 0.25 and 1.8 mg of Varroa-active dsRNA, respectively, for the 1X and 10X Varroa-active dsRNA treatments.

In four other lepidopteran species, diamondback moth (*Plutella xylostella*), legume pod borer (*Maruca vitrata*), spotted stalk borer *(Chilo partellus)*, and tobacco cutworm (*Spodoptera litura*), larvae feeding on fresh plant tissue and provided either 1.2 x 10^−4^ mg ß1 integrin dsRNA or 3 x 10^−3^ mg chitin synthase dsRNA (both dsRNA molecules targeted the individual species’ mRNA) had 50 to 100% mortality [38, 39]. These results suggest that monarch larvae are less sensitive to dsRNA molecules and/or the *v-ATPase* mRNA could be recalcitrant to silencing. Lower levels (ca. 10%) of mortality via V-ATPase silencing were also seen in cotton bollworm (*Helicoverpa armigera)* larvae that were provided 0.01 mg/cm^2^ treated leaves (dose not provided) for 10 days [40]. More data across species and genes are needed to make more conclusive comparisons.

In both tropical and common milkweed bioassays, the majority (55 to 70%) of monarchs that successfully pupated were third-instar larvae on the fourth day of observation; of the remaining monarchs, 95% were fourth instars and 5% were second instars. On Day 8, 67 to 92% of monarchs were fifth instars, and the rest were fourth instars. On Day 12, 83 to 100% of monarchs were pupae, and the rest were fifth instars. There were no differences in larval or pupal developmental time between treatments; the mean number of days it took larvae to pupate ranged from 11.2 to 11.6 days. Previous studies reported a mean neonate to pupal developmental time of about 12 and 13 days for monarch larvae reared at 27 and 25°C, respectively [41, 42]. There were also no differences in pupal weights across treatments in both common and tropical milkweed bioassays. The average pupal weight in the common milkweed bioassays was greater (1176 vs. 970 mg) likely because the larvae were, on average, provided more milkweed leaves than larvae in the tropical milkweed bioassays (Fig 3). Finally, there was no effect of Varroa or monarch-active dsRNA on the eclosion rate across treatments or runs. The average eclosion rates in the common and tropical milkweed runs were 0.93 and 0.97, respectively.

Our results provide evidence that chronic monarch larval exposure to monarch V-ATPase dsRNA has no biologically significant effect on monarch survival, growth, development, or eclosion rates. The results are consistent with Pan et al. [31] who fed first-instar monarchs dsRNA derived from monarch *v-ATPase A* mRNA for two days and then provided the larvae untreated honeyvine milkweed leaves (the first-instar stage lasted 4 to 5 days in this experiment). These researchers observed no effects on survival and overall development time; significant differences in development times for some instars between treatments may have been an artifact of using honeyvine milkweed leaves, which in some cases, can delay larval development [43, 44]. The lack of significant effects observed by Pan et al. [31] could have been due to the abbreviated length of dsRNA exposure, which may have resulted in an internal dose that was insufficient to elicit a toxic response and/or the peak dsRNA internal dose did not correspond to a critical developmental window (e.g., pupation and metamorphosis to the adult). In the present study, we chronically exposed monarch larvae to 0.020 to 0.034 mg/g monarch-active dsRNA milkweed leaf concentration and did not detect an adverse impact on survival, development, growth, or eclosion, as compared to larvae reared on untreated milkweed leaves. These findings are broadly consistent with the conclusions of Terenius et al. [35], who reviewed more than 150 RNAi experiments in the insect order Lepidoptera. The authors reported that the technology seemed particularly efficacious at targeting immune genes in the family Saturniidae (species in the family Nymphalidae, to which monarchs belong, were not studied at the time of review). However, genes from the protein binding group, e.g., V-APTase and calmodulin, were refractory to silencing. Shukla et al. [45] also found that while Lepidopteran cell lines absorbed V-ATPase dsRNA, they did not process it to siRNA, which is necessary for gene silencing.

We are aware of only three chronic studies with Lepidopteran larvae that employed dietary dsRNA exposure methods without a bacterial or polymer vehicle. These studies used dsRNA molecules with a 100% base pair match to the mRNA of the target insect. Choi et al. [46] fed dsRNA encoding the pheromone biosynthesis activating neuropeptide (PBAN) gene to corn earworm (*Helicoverpa zea*) and tobacco budworm (*Heliothis virescens*); treated larvae experienced delayed growth, failed pupal development, and increased mortality. Cotton bollworm larvae that were fed artificially synthesized siRNA that targeted their acetylcholine esterase enzyme had higher mortality, diminished growth, smaller pupal weights, and reduced fecundity compared to control larvae [47]. Whyard et al. [22] found that tobacco hornworm (*Manduca sexta*) larvae that were fed dsRNA targeting their V-ATPase transcripts had a LC_50_ of 0.011 mg/g diet. These three studies employed dsRNA-treated artificial diets rather than treated-host plant leaves. Of note, tobacco cutworm larvae that fed on cabbage leaves had greater dsRNA-degrading activity than larvae that were reared on an artificial diet [36]. These authors suggest that artificial diet could potentially influence dsRNase expression, dsRNA stability, and RNAi efficiency. As our study employed fresh host plant leaves, a comparison of our results with chronic studies that employed an artificial diet may not be appropriate.

The recalcitrant response of monarch larvae also could be due to high gut pH and/or the presence of dsRNases in the gut. For example, RNA is most stable at a pH of 4.0 to 5.0 and lepidopterans have a gut pH greater than 8.0, which suggests dsRNA molecules may be unstable in this environment [48]. In addition, multiple dsRNases have been found in the gut or hemolymph of several lepidopteran larvae, including tobacco cutworm, fall armyworm (*Spodoptera frugiperda*), silkworm (*Bombyx mori*), and tobacco hornworm [36, 48, 49]. If the monarch gut contains ribonucleases, it could further reduce the internal dsRNA dose below a level needed to silence mRNA signaling. Low dietary dsRNA concentrations, combined with high gut pH and dsRNase activity, could be another potential factor responsible the lack dsRNA effects in Lepidoptera. For example, Terenius et al. [35] observed that dietary dsRNA insecticides silenced genes at only high concentrations. We used a 5 mg/mL monarch-active dsRNA suspension in the present study, which represents a practical upper limit of exposure given the solubility of the material. Given these factors, it is not surprising that Lepidopterans demonstrate low sensitivity to dsRNA products, with LC_50_s often exceeding 1.0 mg/g [48, 49].

While our results show that monarch larvae exposed to dsRNA through their diet are unlikely to show adverse effects, application of foliar dsRNA insecticides could result in cuticular exposure. Penetration and absorption of dsRNA through the cuticle could bypass gut nucleases and alkalinity [49]. For example, Lepidoptera Asian corn borer (*Ostrinia furnacalis*) had 100% larval mortality five days after the larvae and their diet were topically sprayed with dsRNA encoding the chymotrypsin-like serine protease C3 gene [50]. Although there are no currently registered foliar dsRNA products, the technology has shown promise and could be further developed in the near future [51]. For example, Miguel and Scott [52] applied a dsRNA derived from Colorado potato beetle (CPB) to leaves of potato plants. CPB larvae feeding on the treated plants had high mortality. They also found that dsRNA was stable for at least 28 days under greenhouse conditions, which indicates long-term exposure to the insecticide is possible. Future commercial production and application of foliar dsRNA insecticides could result in spray drift exposure to non-target organisms near agricultural fields [48], including monarch larvae.

Monarch butterfly populations have declined in the last two decades [53, 54], and the U.S. Fish and Wildlife Services recently listed it as a candidate species under the Endangered Species Act [55]. Other non-target Lepidopteran populations are also declining [56–58]. Effective conservation practices involve understanding risks of pesticides, including new technologies such as dsRNA insecticides. In this regard, our study adds to the growing evidence that some Lepidopteran species may not be adversely impacted by dsRNA products, particularly by those that target protein binding groups.

## Supporting information

S1 Table Preparation of solutions used in the QuantiGene® Singleplex Assay Kit. (DOCX)

S1 Fig Sequence of the Varroa-active dsRNA (Inberg and Mahak 2016). (DOCX)

S2 Fig Varroa-active dsRNA closest predicted sequence match and location in Varroa mite genome. A: Closest predicted sequence match. B: Varroa-active dsRNA (query) overlap in the Varroa mite genome (subject).
(DOCX)

S3 Fig Varroa-active dsRNA comparison to honeybee sequences. A: Varroa-active dsRNA (query) overlaps in honeybee genome (subject). B: Varroa-active dsRNA (query) overlap in the honeybee calmodulin mRNA (subject).
(DOCX)

S4 Fig Varroa-active dsRNA comparison to monarch butterfly sequences. A: Varroa-active dsRNA (query) overlaps in monarch butterfly genome (subject). B: Varroa-active dsRNA (query) overlap in the monarch butterfly calmodulin mRNA (subject).
(DOCX)

S5 Fig Representative QuantiGene calibration curves for monarch-active dsRNA (A) and Varroa-active dsRNA (B). (DOCX)

S6 Fig Correlation between measured leaf concentration and mortality for monarch butterfly (MB) dsRNA, 1X Varroa (VL) dsRNA, and 10X Varroa (VH) dsRNA treatments. Data were analyzed separately for common and tropical milkweed. Each point on the graph indicates a bioassay run.
(DOCX)
