# Supplementary material for: Evaluating toxicity of Varroa mite (Varroa destructor)-active dsRNA to monarch butterfly (Danaus plexippus) larvae
Source: PLoS One. 2021 Jun 2;16(6):e0251884. doi: 10.1371/journal.pone.0251884 (PMC8171953; doi:10.1371/journal.pone.0251884)
Supplement: S1 Table — (DOCX) [file pone.0251884.s001.docx]

S1 Table. Preparation of solutions used in the QuantiGene® Singleplex Assay Kit.

| **Solution** | **Brand/Company** | **Components** | **Notes** |
| --- | --- | --- | --- |
| Sample diluent  (*actually* *used*) | Baker’s Yeast RNA [Lot #:SLBV7182]: *Sigma-Aldrich, St. Louis, MO, USA* | 1:1000 dilution of Baker’s Yeast Solution made with UltraPure™ water [e.g. 100 µl of Baker’s Yeast stock solution and 100 mL of UltraPure™ water]  *(baker’s yeast stock solution: 10 mg of baker’s yeast RNA and 1 mL of UltraPure™ water)* | Keep both stock solution and diluted solution refrigerated |
|  | UltraPure™ water [Ref #:10977-015]: *Invitrogen by Life Technologies, Grand Island, NY, USA* |  |  |
| Working solution | UltraPure™ water [Ref #:10977-015]: *Invitrogen by Life Technologies, Grand Island, NY, USA* | 5.3 mL UltraPure™ Water  3.8 mL lysis mixture*  115 µL blocking reagent* | Vortex 10 sec. Make fresh daily. Makes enough for 1 plate. |
|  | Lysis mixture [Ref #:10093]: *Invitrogen by Thermo Fisher Scientific, Affymetrix Inc., Santa Clara, CA, USA* |  |  |
|  | Blocking reagent [Ref #:13254]: *Invitrogen by Thermo Fisher Scientific, Affymetrix Inc., Santa Clara, CA, USA* |  |  |
| Wash buffer | Buffer component #1 [Ref #:10842]: *Invitrogen by Thermo Fisher Scientific, Affymetrix Inc., Santa Clara, CA, USA* | 1.05 mL buffer component #1*  1.75 mL buffer component #2*  350 mL nuclease-free water | Mix well. Make fresh daily. Makes enough for 1 plate. |
|  | Buffer component #2 [Ref #:10845]: *Invitrogen by Thermo Fisher Scientific, Affymetrix Inc., Santa Clara, CA, USA* |  |  |
|  | Nuclease-free water [CAT #:9153-1]:  *RICCA Chemical Company, Arlington, TX, USA* |  |  |
| Pre-amplifier solution | Amplifier/label probe diluent [Ref #:14539]: *Invitrogen by Thermo Fisher Scientific, Affymetrix Inc., Santa Clara, CA, USA* | 11 mL Amplifier/label probe diluent*  11 µL pre-amplifier reagent* | Vortex 10 sec. Mix prior to use. Makes enough for 1 plate. |
|  | Pre-amplifier reagent [Ref #:15094]: *Invitrogen by Thermo Fisher Scientific, Affymetrix Inc., Santa Clara, CA, USA* |  |  |
| Amplifier solution | Amplifier/label probe diluent [Ref #:14539]: *Invitrogen by Thermo Fisher Scientific, Affymetrix Inc., Santa Clara, CA, USA* | 11 mL Amplifier/label probe diluent*  11 µL amplifier reagent* | Vortex 10 sec. Mix prior to use. Makes enough for 1 plate. |
|  | Amplifier reagent [Ref #:15097]: *Invitrogen by Thermo Fisher Scientific, Affymetrix Inc., Santa Clara, CA, USA* |  |  |
| Label probe Solution | Amplifier/label probe diluent [Ref #:14539]: *Invitrogen by Thermo Fisher Scientific, Affymetrix Inc., Santa Clara, CA, USA* | 11 mL Amplifier/label probe diluent*  11 µL label probe reagent* | Vortex 10 sec. Mix prior to use. Makes enough for 1 plate. |
|  | Label probe reagent [Ref #:10087]: *Invitrogen by Thermo Fisher Scientific, Affymetrix Inc., Santa Clara, CA, USA* |  |  |

*Provided as part of the QuantiGene® 2.0 Singleplex Assay kit.
