# Supplementary figures and images for: Evaluating toxicity of Varroa mite (Varroa destructor)-active dsRNA to monarch butterfly (Danaus plexippus) larvae
Source: PLoS One. 2021 Jun 2;16(6):e0251884. doi: 10.1371/journal.pone.0251884 (PMC8171953; doi:10.1371/journal.pone.0251884)

S1 Fig. Sequence of the Varroa dsRNA (Inberg and Mahak 2016).


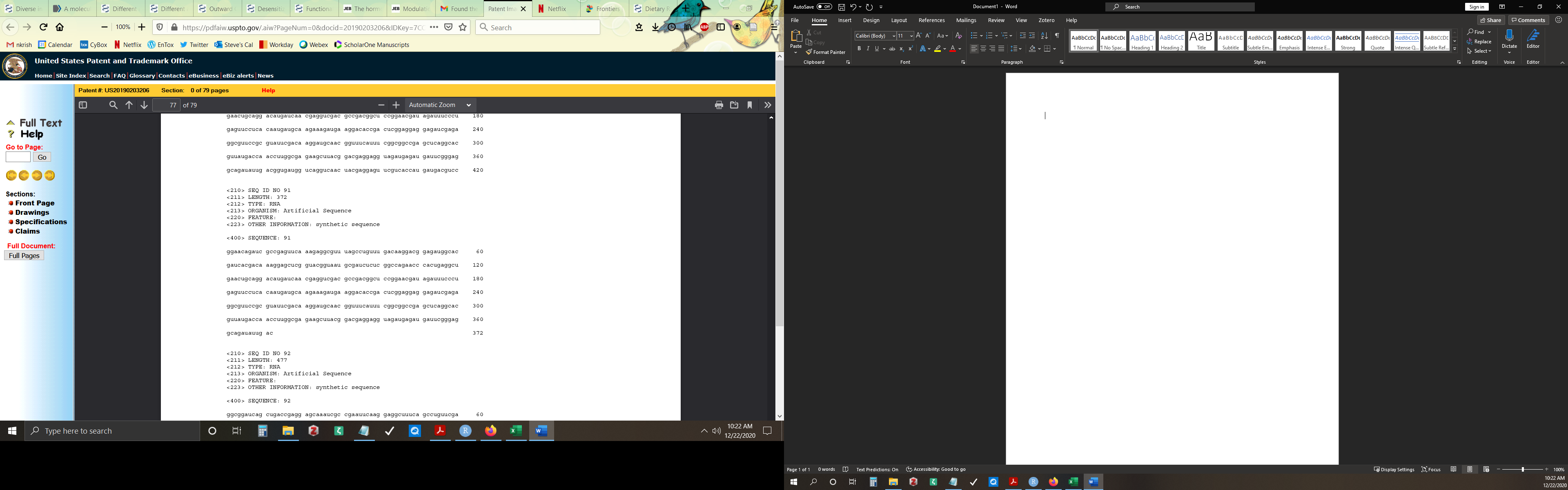

Supplement: S1 Fig — (DOCX) [file pone.0251884.s002.docx]
