# Supplementary material for: Evaluating toxicity of Varroa mite (Varroa destructor)-active dsRNA to monarch butterfly (Danaus plexippus) larvae
Source: PLoS One. 2021 Jun 2;16(6):e0251884. doi: 10.1371/journal.pone.0251884 (PMC8171953; doi:10.1371/journal.pone.0251884)
Supplement: S2 Fig — A: Closest predicted sequence match. B: Varroa-active dsRNA (query) overlap in the Varroa mite genome (subject). (DOCX) [file pone.0251884.s003.docx]

S2 Fig. Varroa dsRNA closest predicted sequence match and location in Varroa mite genome.

1. Closest predicted sequence match.


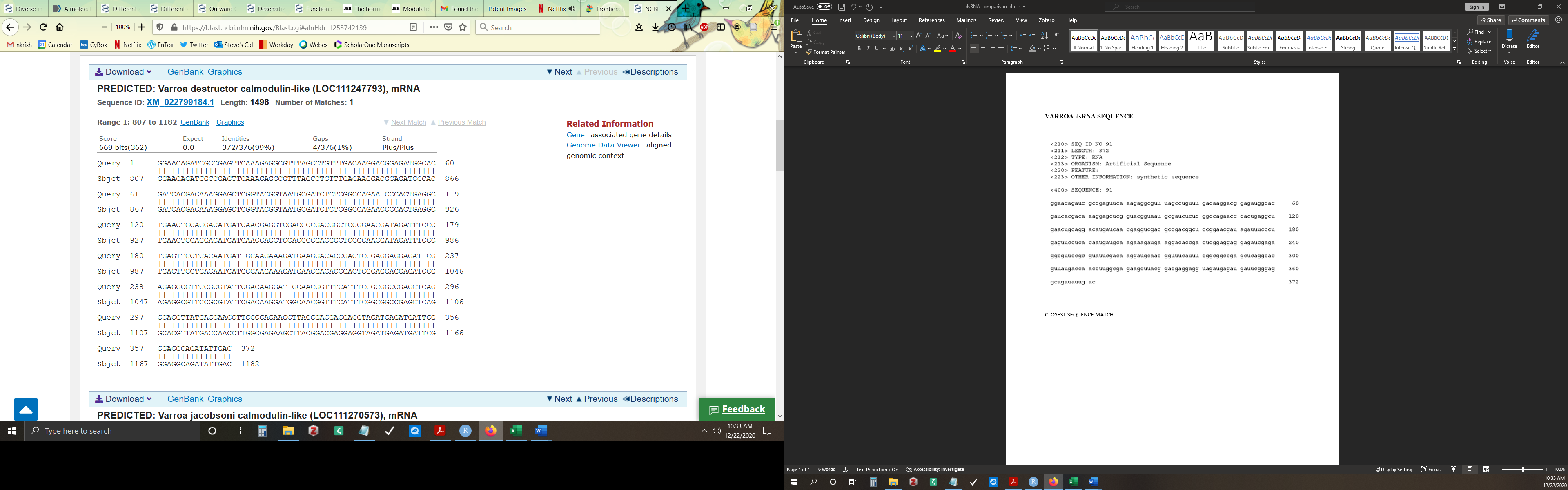


1. Varroa dsRNA (query) overlap in the Varroa mite genome (subject).


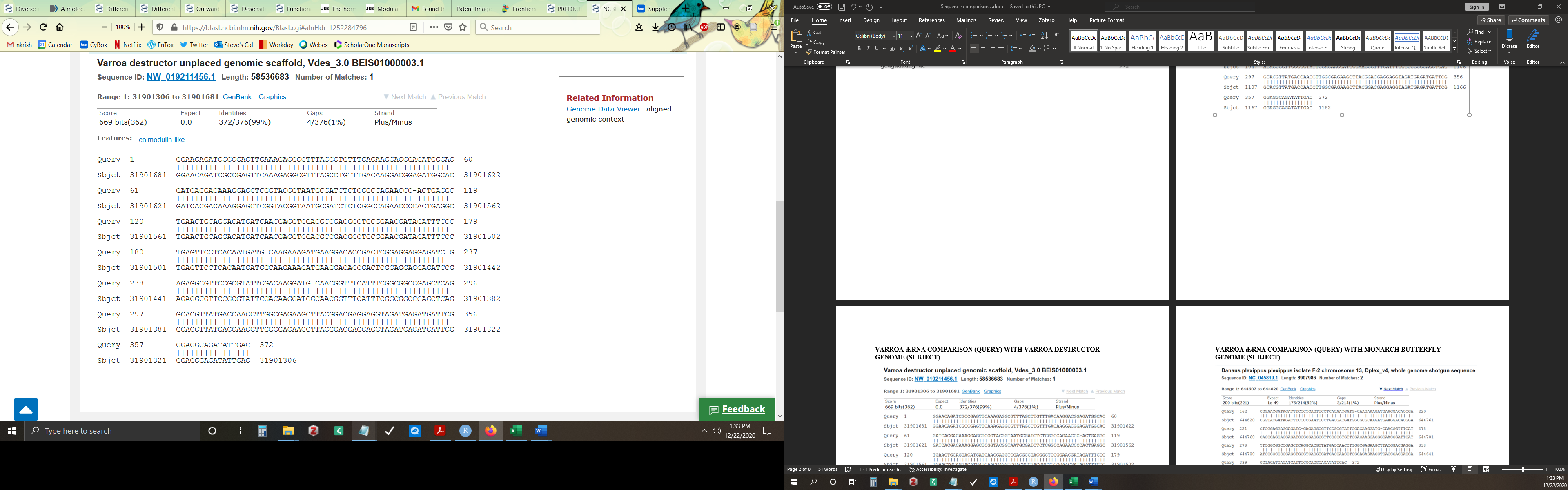


SUMMARY: The closest sequence to the Varroa dsRNA is predicted to be the Varroa mite calmodulin mRNA. The same region of sequence overlap is seen when the Varroa dsRNA sequence is compared to the whole Varroa mite genome.
