# Supplementary material for: Evaluating toxicity of Varroa mite (Varroa destructor)-active dsRNA to monarch butterfly (Danaus plexippus) larvae
Source: PLoS One. 2021 Jun 2;16(6):e0251884. doi: 10.1371/journal.pone.0251884 (PMC8171953; doi:10.1371/journal.pone.0251884)
Supplement: S3 Fig — A: Varroa-active dsRNA (query) overlaps in honeybee genome (subject). B: Varroa-active dsRNA (query) overlap in the honeybee calmodulin mRNA (subject). (DOCX) [file pone.0251884.s004.docx]

S3 Fig. Varroa dsRNA comparison to honeybee sequences.

1. Varroa dsRNA (query) overlaps in honeybee genome (subject)


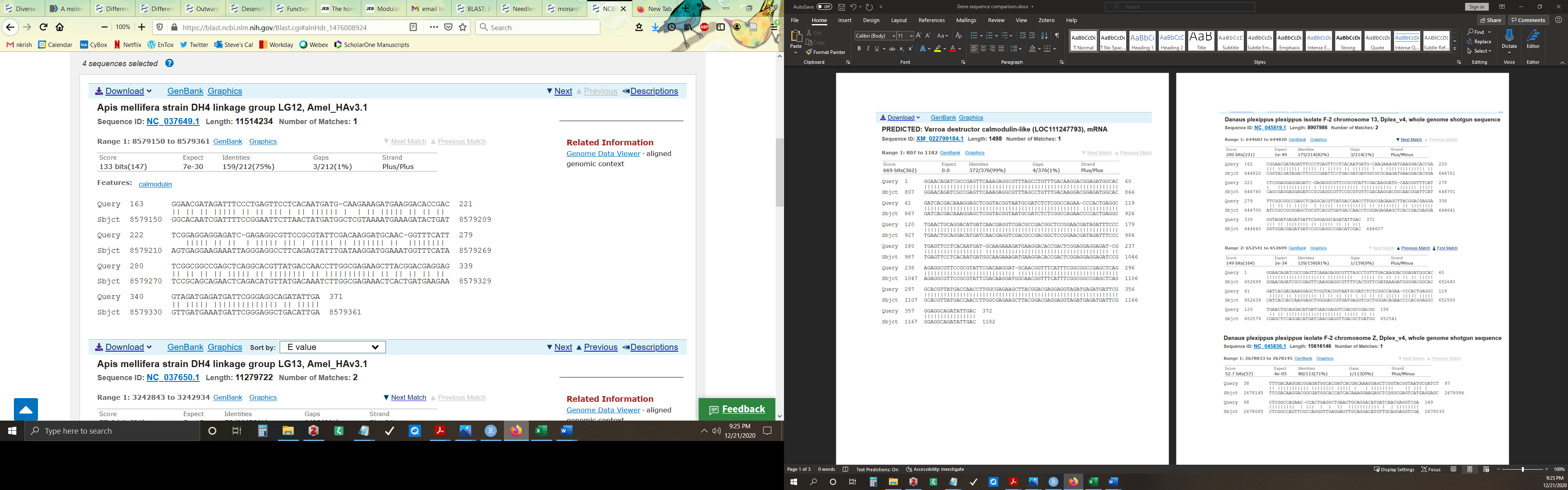


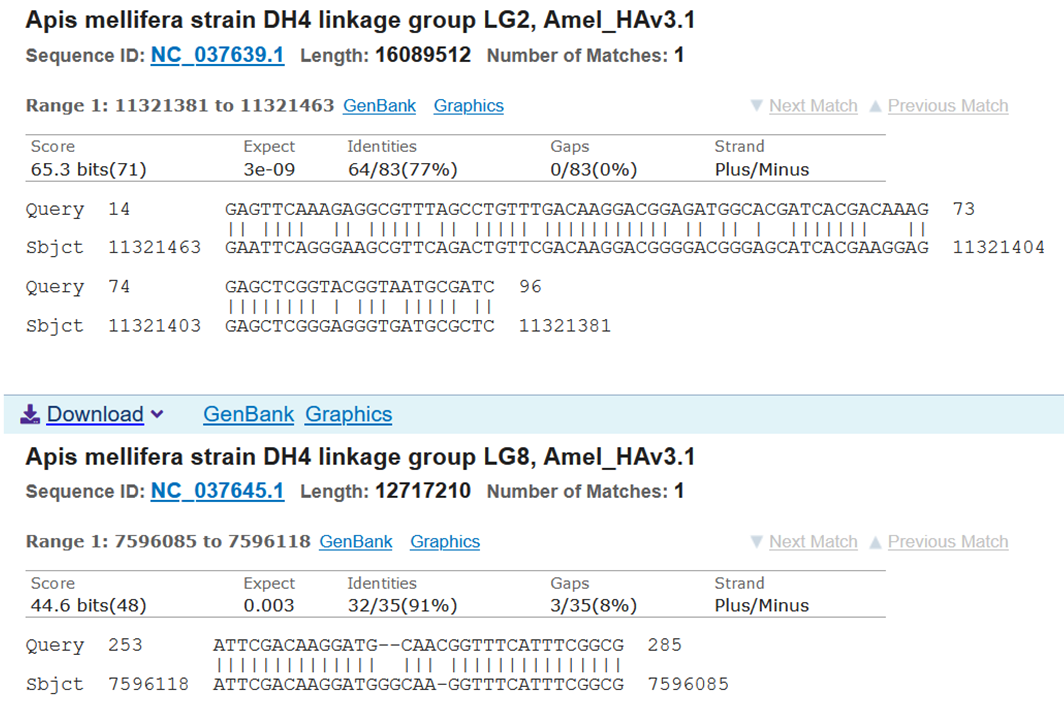


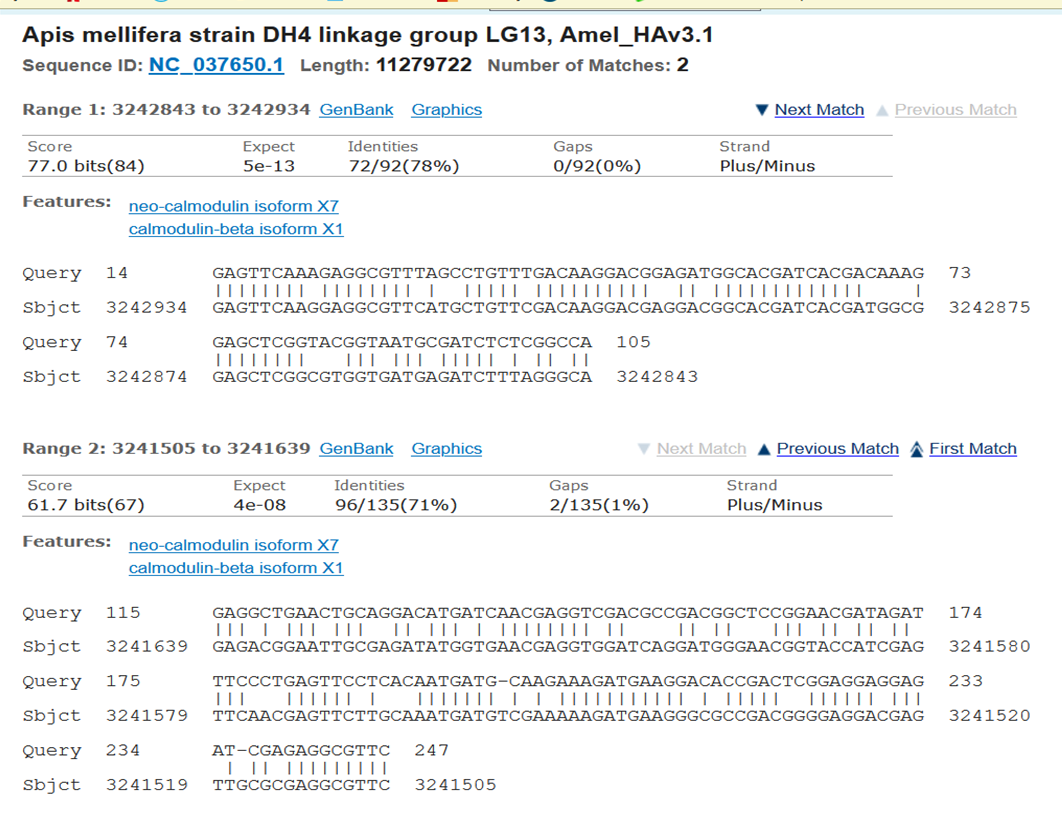


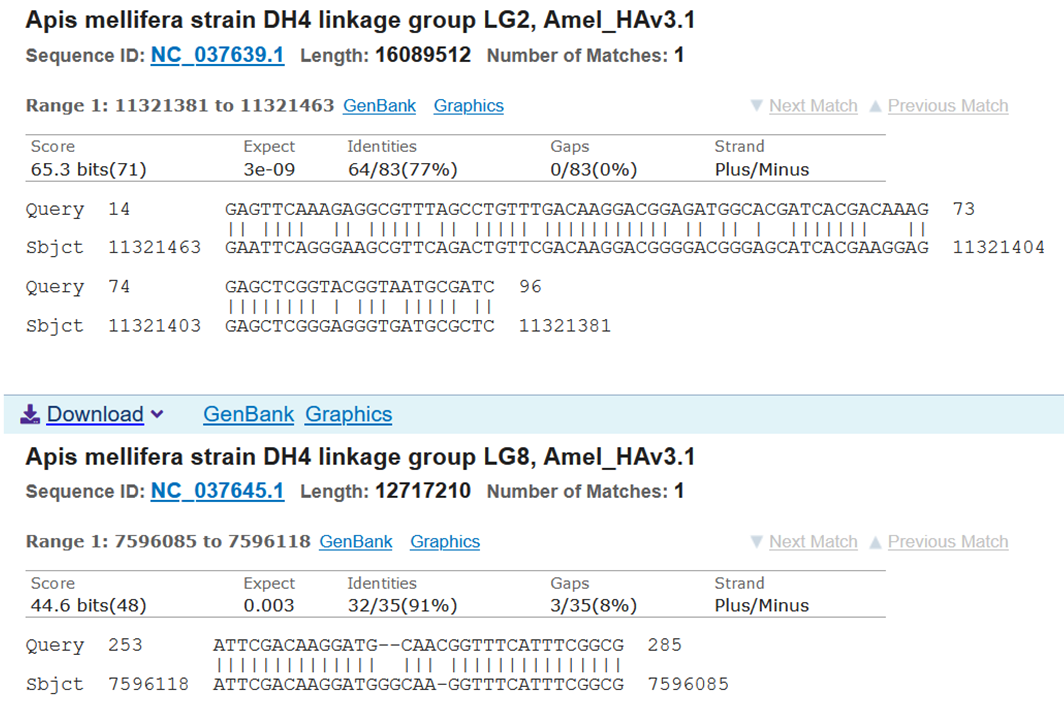


1. Varroa dsRNA (query) overlap in the honeybee calmodulin mRNA (subject).


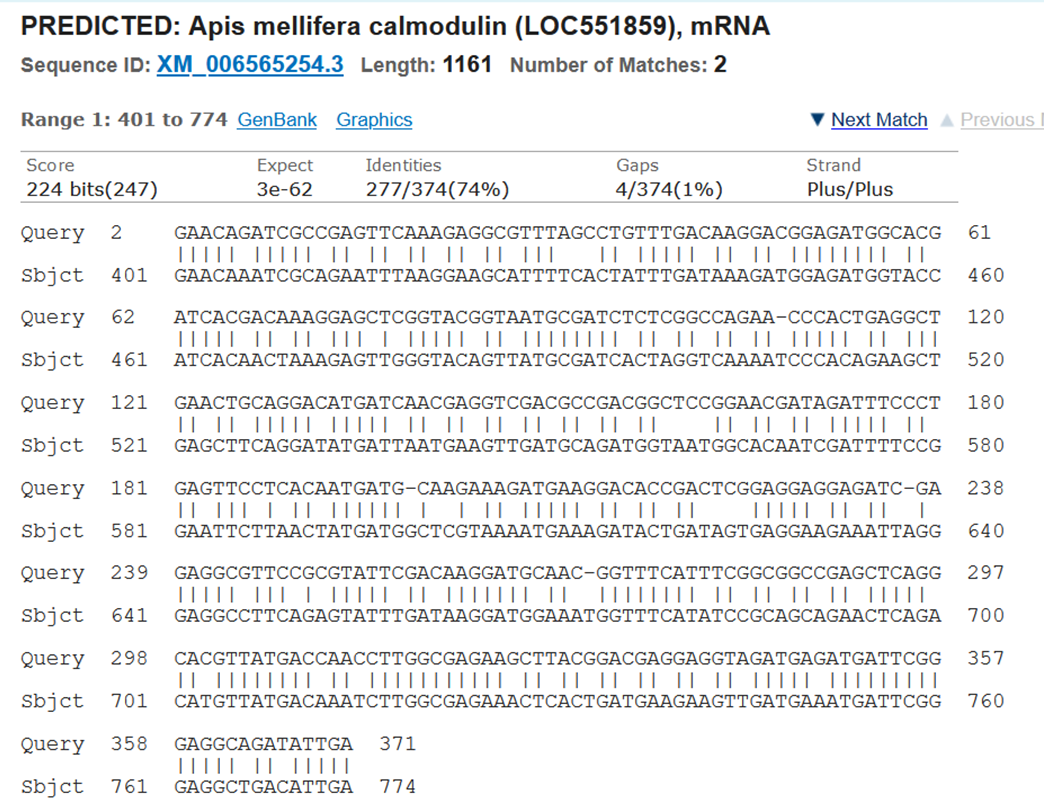


SUMMARY: The Varroa dsRNA has sequence similarity to four regions in the honeybee genome. One of these regions (DH4 linkage group LG12), which contains a shared 14 nucleotide sequence, overlaps with the honeybee calmodulin mRNA. Another region (DH4 linkage group LG8), which contains a shared 15 nucleotide sequence, did not overlap with the honeybee calmodulin mRNA. There are no shared 21 nucleotide sequences.
