# Supplementary material for: Evaluating toxicity of Varroa mite (Varroa destructor)-active dsRNA to monarch butterfly (Danaus plexippus) larvae
Source: PLoS One. 2021 Jun 2;16(6):e0251884. doi: 10.1371/journal.pone.0251884 (PMC8171953; doi:10.1371/journal.pone.0251884)
Supplement: S4 Fig — A: Varroa-active dsRNA (query) overlaps in monarch butterfly genome (subject). B: Varroa-active dsRNA (query) overlap in the monarch butterfly calmodulin mRNA (subject). (DOCX) [file pone.0251884.s005.docx]

S4 Fig. Varroa dsRNA comparison to monarch butterfly sequences.

1. Varroa dsRNA (query) overlaps in monarch butterfly genome (subject)


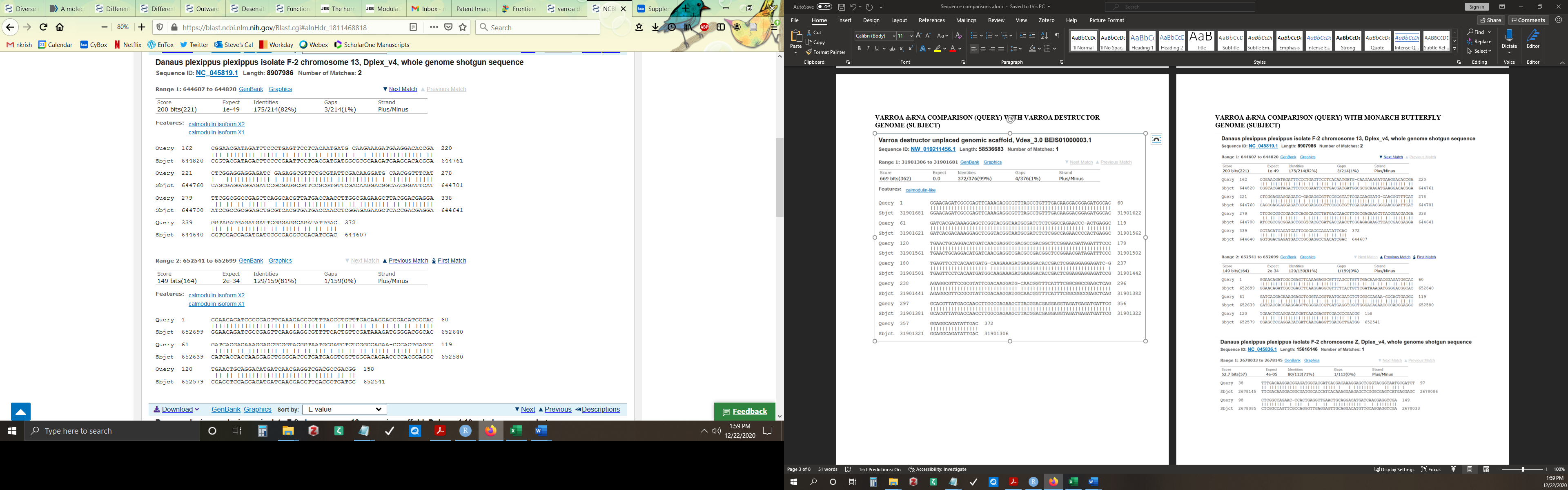


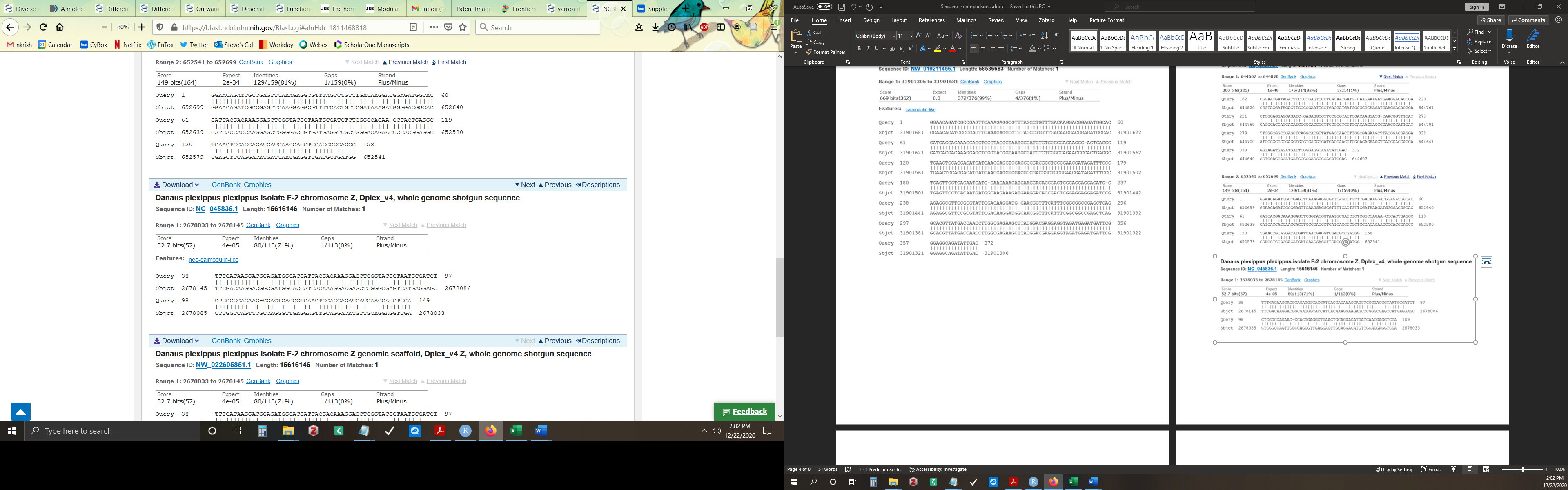


1. Varroa dsRNA (query) overlap in the monarch butterfly calmodulin mRNA (subject).


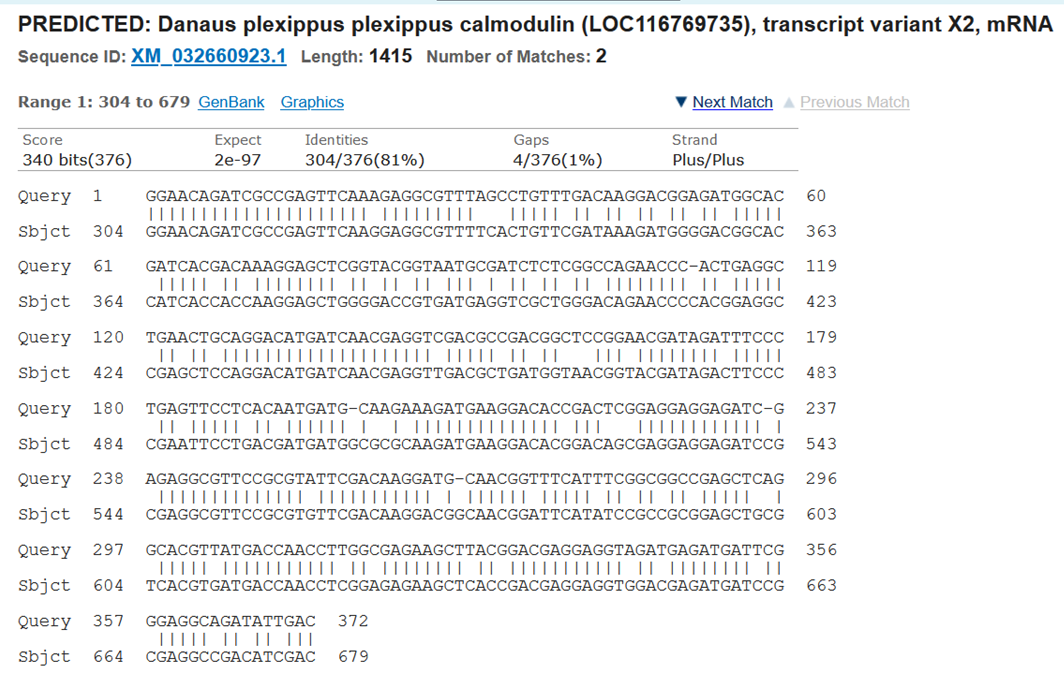
SUMMARY: The Varroa dsRNA has sequence similarity to two regions in the monarch butterfly genome. One of these regions (F-2 chromosome 13), which contains a shared 21 nucleotide sequence, overlaps with the monarch butterfly calmodulin mRNA.
