# Supplementary material for: Evaluating toxicity of Varroa mite (Varroa destructor)-active dsRNA to monarch butterfly (Danaus plexippus) larvae
Source: PLoS One. 2021 Jun 2;16(6):e0251884. doi: 10.1371/journal.pone.0251884 (PMC8171953; doi:10.1371/journal.pone.0251884)
Supplement: S5 Fig — (DOCX) [file pone.0251884.s006.docx]

S5 Fig. Representative QuantiGene calibration curves for monarch dsRNA (A) and Varroa dsRNA (B).
