# Supplementary material for: Evaluating toxicity of Varroa mite (Varroa destructor)-active dsRNA to monarch butterfly (Danaus plexippus) larvae
Source: PLoS One. 2021 Jun 2;16(6):e0251884. doi: 10.1371/journal.pone.0251884 (PMC8171953; doi:10.1371/journal.pone.0251884)

S6 Fig. Correlation between measured leaf concentration and mortality for monarch butterfly (MB) dsRNA, 1X Varroa (VL) dsRNA, and 10X Varroa (VH) dsRNA treatments. Data were analyzed separately for common and tropical milkweed. Each point on the graph indicates a bioassay run.


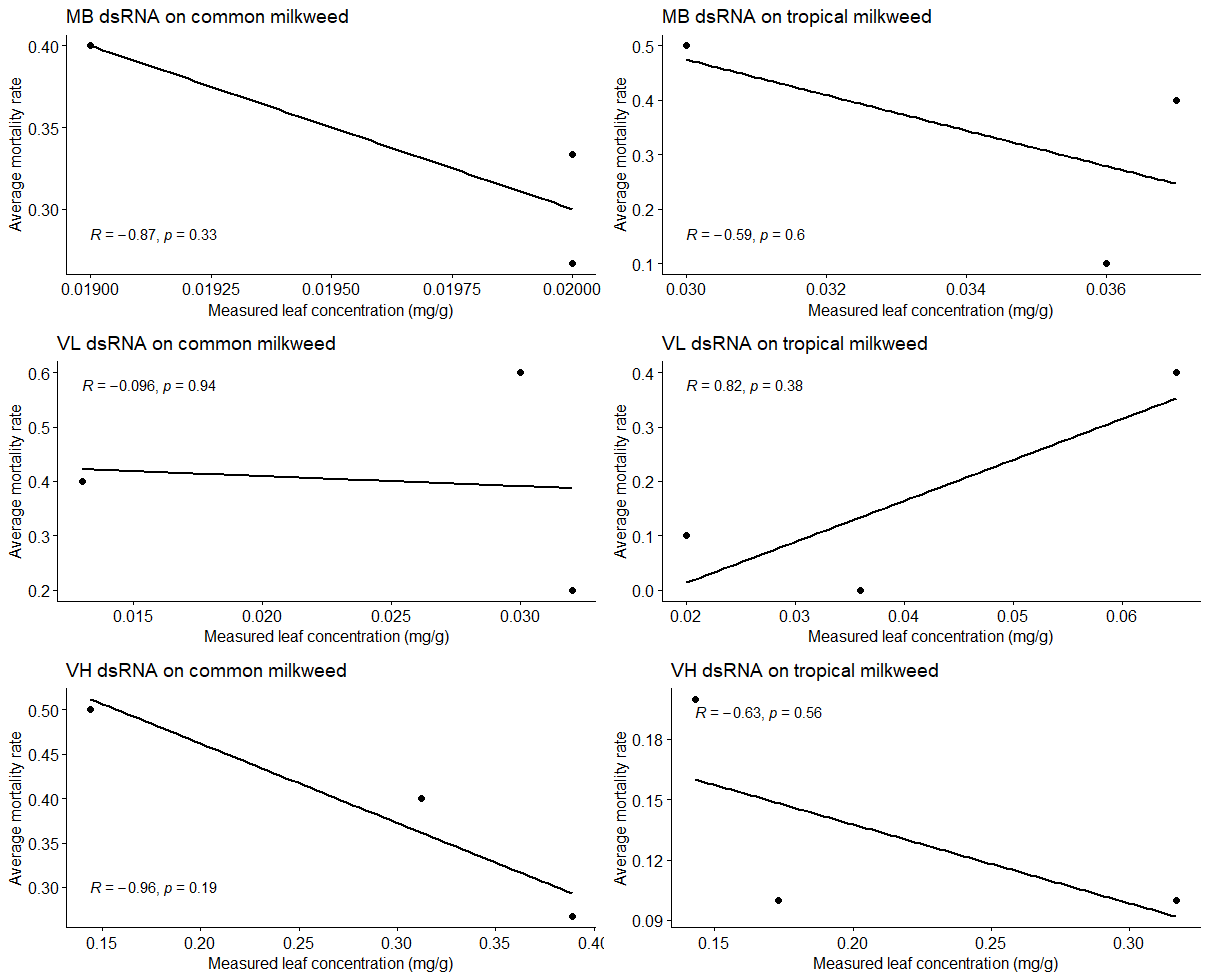

Supplement: S6 Fig — Data were analyzed separately for common and tropical milkweed. Each point on the graph indicates a bioassay run. (DOCX) [file pone.0251884.s007.docx]
